# Origin and Regenerative Potential of Vertebrate Mechanoreceptor-Associated Stem Cells

**DOI:** 10.1155/2012/837626

**Published:** 2012-10-02

**Authors:** Darius Widera, Stefan Hauser, Christian Kaltschmidt, Barbara Kaltschmidt

**Affiliations:** ^1^Department of Cell Biology, University of Bielefeld, Universitätsstraße 25, 33501 Bielefeld, Germany; ^2^Department of Molecular Neurobiology, University of Bielefeld, Universitätsstraße 25, 33501 Bielefeld, Germany

## Abstract

Meissner corpuscles and Merkel cell neurite complexes are highly specialized mechanoreceptors present in the hairy and glabrous skin, as well as in different types of mucosa. Several reports suggest that after injury, such as after nerve crush, freeze injury, or dissection of the nerve, they are able to regenerate, particularly including reinnervation and repopulation of the mechanoreceptors by Schwann cells. However, little is known about mammalian cells responsible for these regenerative processes. Here we review cellular origin of this plasticity in the light of newly described adult neural crest-derived stem cell populations. We also discuss further potential multipotent stem cell populations with the ability to regenerate disrupted innervation and to functionally recover the mechanoreceptors. These capabilities are discussed as in context to cellularly reprogrammed Schwann cells and tissue resident adult mesenchymal stem cells.

## 1. Introduction

**Figure 1:**
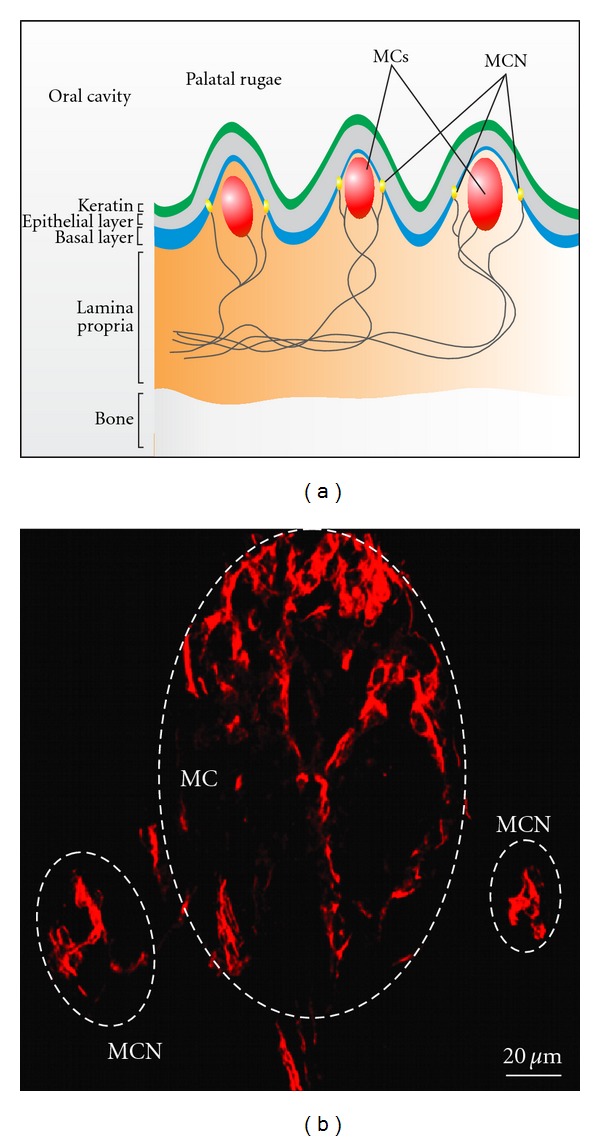
Anatomical localization of Meissner corpuscles (MCs) and Merkel cell-neurite complexes (MCN) within rodent hard palate. (a) MCs are located centrally within palatal rugae in the lamina propria, whereas MCN can be found within the basal layer. (b) Nestin expression within rat palatal MCs and adjacent to MCN. Cryosections of rat hard palate were stained with mouse anti-Nestin antibody (clone Rat401) followed by incubation with secondary Alexa555-coupled anti-mouse detection antibody. Confocal analysis revealed strong immunoreactivity in numerous cells within MCs and adjacent to MCN.

**Figure 2:**
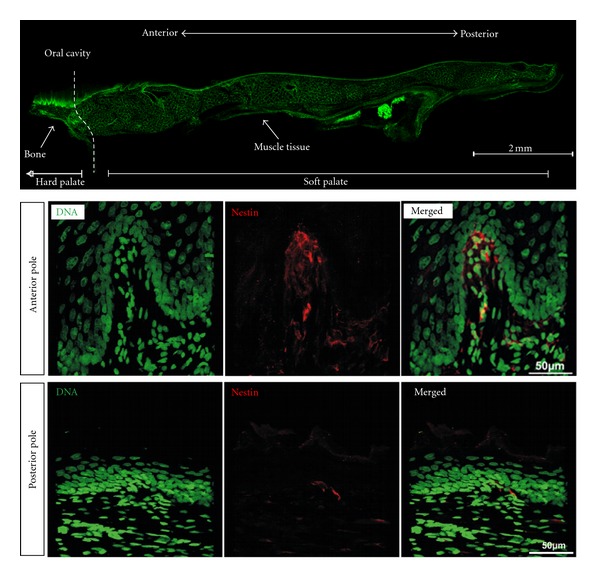
Anterior-posterior gradient of cells Nestin expressing within the rat palate. Cryosections of rat palate were stained with mouse anti-Nestin antibody (clone Rat401) followed by Alexa 555 coupled detection antibody and nuclear counterstaining using Sytox Green. Confocal analysis revealed the presence of numerous Nestin-positive MCs and Merkel cell-neurite complexes within the hard palate (anterior pole), whereas within the soft palate (posterior pole) only few Merkel cell neurites complexes and nearly no MCs were detected.

Meissner corpuscles (MCs, also called tactile corpuscles) were first described in 1852 by the German physiologists Rudolf Wagner and Georg Meissner [1]. These are encapsulated, rapidly adapting mechanoreceptors responsible for sensing light touch on the skin. Recently, due to their immunocytochemical properties, it has been proposed that MC may also act as nociceptors [2]. They can be found within the dermis, beneath the basal layer of skin regions sensitive to light touch. Within the murine, rat and human palatal mucosa, MCs are located centrally within palatal ridges (*rugae palatinae*) and are often accompanied by Merkel cell-neurites [3] (see Figure 1(a)). Remarkably, an anterior-posterior gradient of Nestin-expressing cells within the rat palate could be identified (see Figure 2). In particular, numerous Nestin-positive MCs can be observed in the lamina propria of hard palate, whereas nearly no MCs are present in the soft palate. In humans, the number of MCs gradually decreases with age [4]. 

A further type of highly specialized mechanoreceptors is the Merkel cell-neurite complexes (Merkel nerve endings), which are, in contrast to Meissner corpuscles, not encapsulated and seem to act as slowly adapting mechanoreceptors responsible for sustained sensing of mechanical pressure. In mammals, they are widely distributed and can be found in the basal layer of the palatal/oral mucosa (see Figure 1(a)), as well as in hairy and glabrous skin. Merkel cells were first described in 1875 by Friedrich Siegmund Merkel [5] and were originally termed *“Tastzellen”* (German: *touch cells*) (reviewed in [6]). 

Remarkably, after injury, such as experimental nerve crash or freeze injury, MCs and Merkel cells seem to harbor a limited capacity to regenerate [7–9]. However, the exact cellular and developmental origin of plastic cells within MCs and Merkel cell-neurites responsible for this limited plasticity remains unclear. Recently, we demonstrated high expression of neural crest and general stem cell markers as well as pluripotency-associated transcripts within rat palatal mucosa. In addition, immunocytochemical analysis revealed high expression of Nestin within the MCs and adjacent to Merkel cell-neurite complexes, suggesting the presence of stem cells or other cells with progenitor properties within these mechanoreceptors [3]. 

In the following, we review the cellular composition of Meissner corpuscles and Merkel cell-neurite complexes. We focus on their developmental ancestry, marker expression, and the potential origin of multipotent stem cells within these highly specialized mechanoreceptors, factors which might explain their regenerative potential.

## 2. Cellular Composition of Meissner Corpuscles and Merkel Cell-Neurite Complexes

At the cellular level, MCs consist of a coiled arrangement of endings from up to six myelinated axons that terminate between layers of very thin, flattened, specialized Schwann cells called lamellar cells (see schematic diagram in Figure 4) [10–13]. MCs are connected with thin axons and contain the myelinated axons as well as several unmyelinated nerve fibers [10, 14]. 

MCs contain several fibroblastoid cells, generating the connective tissue capsule. This capsule is distinguishable from the surrounding tissue, since this extracellular matrix is mainly of fibrillar connective tissue consisting of fibrillary proteins like collagens and fibronectins. Importantly, lamellar cells itself are embedded within the so-called basal lamina consisting among others of laminin and collagen type IV (reviewed in [15]). Notably, the extracellular matrix ECM, including the basal lamina, plays an essential role in connection, support, and growth regulation of cells *in vivo *and may therefore have major impact on cells responsible for regenerative processes. This may be due to direct influence on differentiation and proliferation or due to guidance of cells migrating into lesioned MCs.

However, although the acellular components and the cellular composition of MCs have been studied intensely using morphological and immunocytochemical analyses, the existence of further cell types like adult neural crest-derived stem cells or tissue resident mesenchymal stem cells has not been previously reported.

Merkel cell-neurite complexes consist of large, oval Merkel cells (Merkel-Ranvier Cells), which are in synapse-like contact with flat terminal endings of myelinated nerve fibers. In addition to their postulated function as receptor cells essential for tactile discrimination, Merkel cells may also exhibit an endocrine function, as they contain dense core granules comprising various neuropeptides [16–18]. In addition, due to the close spatial proximity of Merkel cells to associated myelinated nerve fibres, several myelinating Schwann cells are known to be located adjacent to Merkel cell-neurite complexes. 

## 3. Expression Pattern

Within MCs, neuronal cells can be identified based on their characteristic expression of neuronal markers including neurofilaments, *β*-III-tubulin, synaptophysin, protein gene product 9.5 (PGP 9.5) [19], and neuron-specific enolase (NSE) [3, 13, 20, 21]. In addition, MCs expressed a panel of specific ion channels (reviewed in [22]). Nonneuronal cells of MC show robust expression of S100, Nestin (Figure 1(b)), Vimentin as well as the epidermal growth factor receptor (EGFR) [3, 20, 21, 23]. Several studies reported strong immunoreactivity for the neurotrophin receptors TrkA, TrkB, and p75^ NTR ^ [20, 21, 24]. Human MCs are negative for cytokeratins and GFAP, as described by Vega and colleagues [20]. Very recently, Meissner corpuscles have been described to express acid-sensing ion channels 2 (ASIC2) [25].

Merkel cells are characterized by the expression of cytokeratins CK8, CK18, CK19, and CK20 (reviewed in [6]) and the absence of CK4 and CK13 [26]. CK20 is of note as the most specific marker of Merkel cells; its expression shows no overlap with other cell types. Moreover, Merkel cells show expression of p75^NTR^, whereas no expression of Vimentin or GFAP can be detected by immunocytochemistry [27, 28]. Although we detected Nestin-positive cells in close proximity to Merkel cell-neurite complexes (Figure 1(b)), there are no direct evidences that Merkel cells itself are positive for this intermediate filament [3]. Indeed, a study by Eispert suggested that Nestin is absent in human Merkel cells of different anatomical origin [29]. Merkel cells are known to express a variety of neuronal markers like NSE, Synaptophysin, and PGP 9.5, whereas Neurofilament is absent [26, 28, 30–33]. Additionally, it was reported that Neurotrophin-3 (NT-3) is required for the development of Merkel cells [34]. Recently, it has been reported that CK20-positive Merkel cells within human skin express Sox2—a pluripotency-associated transcription factor which is crucial for embryonic development as well as embryonic and adult neural crest-derived stem cell populations [35].

## 4. Developmental Origin of Meissner Corpuscles and Merkel Cell-Neurite Complexes

It has been suggested that in addition to the Schwann cell-related cells of the MC, the nerve fibres within NC are also of neural crest origin [36]. In this study, the authors used lineage tracing in Wnt1^Cre^/Ret^fCFP^ mice and demonstrated expression of CFP in neuronal cells, thus elucidating their neural crest ancestry. However, the discussion surrounding the developmental origin of Merkel cells is controversial. In the avian system, it has been suggested that Merkel cells also arise from the neural crest [37]. To address the question of the ontogenic origin of mammalian Merkel cells, Maya Sieber-Blums and colleagues used Wnt1^Cre^/Rosa26R reporter mice in which cells of neural crest lineage can be clearly traced back via expression of *β*-galactosidase (*β*-gal) [38]. In this study, CK8-immunopositive Merkel cells showed clear coexpression of *β*-gal, suggesting their neural-crest origin. In addition, electron microscopy after peroxidase immunostaining revealed presence of *β*-gal and Merkel cell typical dense core granules. A contrary model for the developmental origin of mammalian cells was proposed in 2009 by deletion of Atoh1 in either epidermal (Krt14^Cre ^) or neural crest deleter mice (Wnt1^Cre ^). In this approach, the authors selectively deleted Atoh1, which is necessary for the survival of Merkel cells, in epidermal, Krt14-expressing cells or in Wnt1-positive progeny of the neural crest. Interestingly, epidermal deletion of Atoh1 resulted in loss of Merkel cells in the skin, whereas neural crest-specific deletion showed no significant effects [39]. As the debate surrounding these findings continues, the final assessment of the developmental origin of Merkel cells requires further experimental analysis and as the exact nature of this origin remains elusive.

## 5. Regenerative Potential of MCs and Merkel Cell-Neurite Complexes

In the last two decades of the twentieth century, several reports provided evidences for regenerative capacity of MCs and Merkel cell-neurite complexes [7–9, 40–42]. Through transection of the nerve innervating Meissner corpuscles in mice, Chizuka Ide observed that after an initial degeneration, at least some corpuscles start to regenerate, including re-innervation 30 days after the section and proper morphological appearance 4 months after the surgery [40]. After freezing injury, complete disintegration of cells within the corpuscles occurs after 25 days. In an advanced experimental procedure involving freezing damage, it was observed that the regenerated axons demonstrated extensive branching and morphologically specialized axonal terminals as well as the occurrence of newly formed lamellar cells [41]. Here, the author postulated that Schwann cells migrated into the site of the lesion and differentiated into lamellar cells, although the ancestry of the regenerated axon was not investigated further. Studies by Zelena and colleagues revealed that the regeneration potential of Meissner corpuscles after nerve crush and experimental freeze injury is higher in older animals than in young rats [8, 9].

In addition to MCs, Merkel cells as well seem to regenerate after injury. In an intriguing initial study, reinnervation and an increase of the Merkel cell number after experimental nerve crush were observed 40–100 days after the surgery [7, 43]. Importantly, the authors were able to show that the newly formed Merkel cells appeared physiologically normal and demonstrated typical histological features via electron microscopy and toluidine blue staining.

Although these reports clearly suggested that mechanoreceptors such as MCs and Merkel cell-neurite complexes can regenerate at least to a certain degree, the exact cellular origin of such unexpected plasticity remains unclear. Thus, the existence of so far unknown plastic progenitors or stem cells responsible for the regeneration process seems likely and is further discussed here.

## 6. Potential Stem Cell/Progenitor Populations within MCs and Merkel Cell-Neurite Complexes

### 6.1. Mammalian Adult Neural Crest-Derived Stem Cells

In recent years, several studies revealed the persistence of neural crest-derived stem cells in adult mammals [3, 44–51]. 

In general, such neural crest-derived stem cells (NCSCs) are pluripotent in the early embryonic development (able to differentiate into cells of all germ layers) and become more restricted after migration from their niche between the ectoderm and the neural tube. A limited number of NCSCs that persist in the adult are able to generate multiple cell progenies *in vitro* and *in vivo* and aretherefore, multipotent stem cells (reviewed in [52]). In particular, such cells have been described to efficiently differentiate into neuronal and glial cells, osteogenic cell types, adipocytes, and chondrocytes, as well as into melanocytes and muscle cells. Such adult NCSCs express *in vitro *and* in vivo* high levels of Nestin, which is an intermediate filament originally described in Schwann cells and important for the self-renewal of neural stem cells [53, 54]. Adult NCSCs show expression of Vimentin, Sox2, and, depending on the cultivation method, the neurotrophin receptor p75^NTR^. As observed in adult human NCSCs isolated from respiratory mucosa, such cells also express TrkA (Hauser et al., unpublished observation). In addition, their expression pattern further includes Sox9, Sox10, Klf4, c-Myc, and Oct4 (see [52] for the full list). When cultivated under serum-free conditions *in vitro*, NCSCs form self-adherent clusters similar to neural stem cell-derived neurospheres.

**Figure 3:**
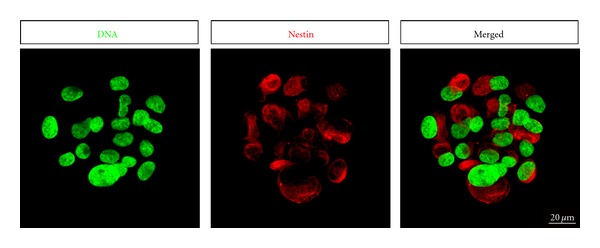
Cultivated palatal neural crest-derived stem cells form self-adherent neurospheres and express the intermediate filament Nestin. Human palatal NCSCs were isolated according to protocol described in [3], fixed using 4% paraformaldehyde and stained using primary antibody against human Nestin and Alexa 555-coupled secondary antibody. DNA was stained using SYTOX green (bar 20 *μ*m).

In 2009, we reported Nestin-expressing NCSCs adjacent to MCs and Merkel cell-neurite complexes within the palatal ridges (*palatal rugae/rugae palatinae*) of adult rats [3]. Due to their anatomical origin within the palate, we termed these palatal neural crest-derived stem cells (pNCSCs). In their niche, the palatal ridges of hard palate, pNCSCs demonstrated high expression of Nestin, as demonstrated by immunocytochemical analysis and RT-PCR. In particular, Nestin immunoreactivity was detected in the periphery, centre, and on the top of Meissner corpuscles, as well as in close proximity to Merkel cell-neurite complexes within the basal layer of the palatal mucosa. We were further able to successfully isolate and expand rat and human palatal NCSCs *in vitro* as free-floating neurosphere cultures [3, 21] (see also Figure 3). Such cultivated pNCSCs were positive for a set of stem cell markers including Nestin, p75^NTR^, Sox9, Notch1, Slug and Snail in addition to Sox2, Klf4, Oct4, and c-Myc. Using appropriate differentiation protocols we demonstrated that pNCSCs were not only able to differentiate into GFAP-expressing glial cells, but also into *β*-III-tubulin, NF- and Map2-positive neuronal cells. Due to such tremendous plasticity, tissue-resistant NCSCs are strong candidates for the observed *in vivo *regeneration of MCs and Merkel cell-neurite complexes after dissection and consequent degeneration. However, at least in case of regeneration after freeze injury, in which all cells within the mechanoreceptors including adjacent cells perish, there must be a further source of plastic cells capable of migration to the site of the lesion. These may be other NCSCs attracted by injury signals (e.g., chemokines and growth factors) from greater spatial distances or different cell types like cellularly reprogrammed Schwann cells or their progenitors (see the following). 

### 6.2. Schwann Cell Progenitors and Dedifferentiated Schwann Cells 

Schwann cells are directly related to the neural crest and share several markers with NCSCs (e.g., Vimentin, Nestin, or p75^NTR^, reviewed in [52]). Schwann cell progenitors (SCPs) are considered as late NCSCs that have established contact with axons [55]. It has been proposed that Schwann cell progenitors and Schwann cells have an extraordinarily unstable phenotype as differentiated Schwann cells can be switched back into more primitive cell types by injury signals or by appropriate cultivation methods [56, 57]. Consequently, these data suggest that Schwann cells and their immature ancestors—Schwann cell progenitors—may act as stem cells. In accordance with this hypothesis, Schwann cells have been described to perform differentiation into melanocytes after the lesion of the adult sciatic nerve [58, 59]. After injury, Schwann cells can reenter the cell cycle and dedifferentiate [56, 60]. Dupin and colleagues demonstrated differentiation of Schwann cells into myofibroblasts, indicating stemness characteristics of such cells *in vitro* [57]. 

Recently, we demonstrated successful cellular reprogramming of adult myelinating Schwann cells into an immature multipotent NCSC phenotype [21]. After isolation and expansion of Schwann cells under culture conditions mimicking an *in vivo* injury, we observed significantly elevated expression levels of p75, c-Myc, Sox2, Klf4, Oct4, Sox9, and Slug. Importantly, we were also able to differentiate such cultivated adult Schwann cells into ectodermal and mesodermal progeny. Such cellular reprogramming into immature neural crest-like phenotype also seems to occur *in vivo* in response to injury. This has been impressively demonstrated in a Wnt^ Cre^/lox-EGFP mouse model [61]. After injury, mature Schwann cells residing at the nerve roots dedifferentiate into proliferating p75^NTR^-positive immature Schwann cells, which migrate into the lesion site. 

It might be assumed that a similar injury-induced reprogramming mechanism could switch mechanoreceptor-associated Schwann cells, such as lamellar cells of MCs, into more primitive phenotype. Since mechanoreceptor-associated, subcutaneous Schwann cells are permanently exposed to mild mechanical stress, a cellular turnover and a latent cellular plasticity may be a hallmark of those cells. Indeed, in our study we detected proliferating, Ki67-positive cells not only in the basal cell layer of the palatal mucosa, but also in the center of MCs [21]. The expression of p75^NTR^ is usually observed in immature, plastic Schwann cell progenitors. However, we and others reported that lamellar cells within MCs show strong p75^NTR^-immunoreactivity in addition to the well-described expression of S100 [20, 21]. Within palatal MCs, S100-immunoreactivity was detected in 100% of the cells in the investigated region, whereas not all cells expressed p75^NTR^ (~50%) [21]. In addition, we observed a high degree of co-expression between Nestin and p75^NTR^ (nearly 100%). Such co-expression of Nestin and p75^NTR^ is a typical marker of immature cells such as early Schwann cell progenitors. However, the most likely source of multipotent neural crest-related stem cells within palatal MCs may be myelinating Schwann cells and not Schwann cell progenitors. Although SCPs are defined as neural crest stem cells which have established contact to nerve fibers, myelinization seems to be crucial for Nestin expression as Nestin has always been observed within myelinating Schwann cells and not in Schwann cells directly in contact with unmyelinated axons [21]. 

### 6.3. Tissue Resident Mesenchymal Stem Cells as Further Putative Stem Cell Type Responsible for Regeneration 

A further adult stem cell type is represented by mesenchymal stem cells. Beside their initially described niche within the mammalian bone marrow, the presence of adult MSCs has also been confirmed in almost all tissues including muscle, fat, and the dermis of diverse types of mucosa [62]. Similar to adult NCSCs, MSCs are able to generate osteogenic, adipogenic, and chondrogenic progeny, although their neuronal differentiation potential is a subject of an ongoing scientific debate [63–65]. At least *in vitro*, MSCs seem to show the ability to differentiate into neuron-like and Schwann cell-like phenotypes [66, 67] (reviewed in [65]). Concerning their morphological and immunocytochemical properties, as well as the expression profile, MSCs show high degree of similarity to fibroblasts, making them nearly indistinguishable *in vivo* [68–71]. Importantly, in addition to typical MSC markers such as CD105, CD73 and CD90, MSCs express also Nestin and Vimentin [64, 72, 73].

As discussed previously, MCs contain not only glial cell, such as lamellar cells, but also fibroblast-like cells which, in the classical view, generate the connective encapsulation of the corpuscle. Remarkably, we observed Nestin immunoreactivity not only in lamellar cells of MCs, but also adjacent to the connective encapsulation of the corpuscles [3, 21]. Therefore, it cannot be excluded that MCs and adjacency of Merkel cell-neurite complexes contain both at least some tissue resident MSCs. 

Taking into consideration that MSCs may be able to differentiate into multiple progenies, also this cell type can address the question of mechanoreceptor regeneration.

In this context, it is noteworthy that in our studies, we focused on cranial mucosa, a tissue where also tissue-resident MSCs are clearly of neural crest origin [74]. Thus, such cranial neural crest-derived MSCs may reveal a higher degree of plasticity than their trunk counterparts, which are not of neural crest ancestry [74].

## 7. Conclusions

**Figure 4:**
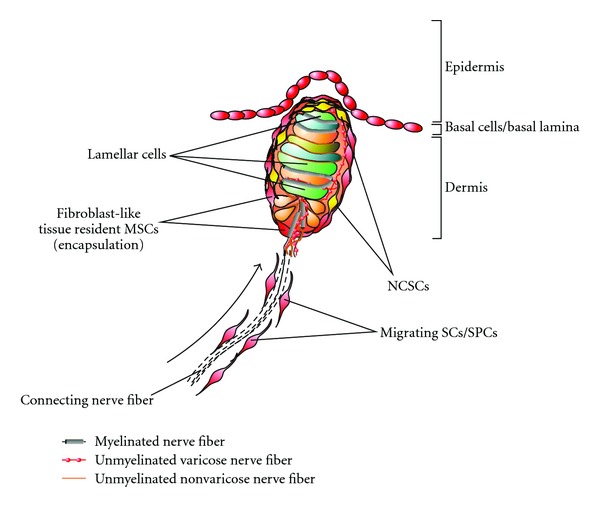
Putative multipotent stem cell/progenitor populations within Meissner corpuscles. After lesion several cell populations may participate to the regeneration process. Firstly, multipotent adult neural crest-derived stem cells NCSCs may directly contribute to the regeneration process. Secondly, lamellar cells and other Schwann cells may be cellularly reprogrammed and re-enter the cell cycle after lesion. This process may be accompanied by migration of Schwann cells and their progenitors from the periphery to the site of the lesion. Finally, tissue resident fibroblast-like MSCs may also play an important role in recovery of mechanoreceptors.

As summarized previously, there are different multipotent cell populations in anatomical adjacency of mechanoreceptors which may be responsible for regenerative processes after lesion (see Figure 4). Firstly, due to their extraordinarily high plasticity, NCSCs may directly contribute to the regeneration process *in vivo. *A second plastic cell population is represented by cellularly reprogrammed Schwann cells, which reenter the cell cycle and dedifferentiate into more primitive phenotypes after lesion in their endogenous niche. Tissue resident MSCs may also play an important role in recovery after degeneration of mechanoreceptors. Additionally, a cooperative manner action, including more than one cell type, cannot be excluded at this time.

A definitive identification of the cellular base of regeneration remains a future challenge, the elucidation of which may help to develop effective treatments for acute injuries and age-related diseases accompanied by loss of mechanoreceptors, such as peripheral neuropathies. *In vitro* cultivation of autologous, mechanoreceptor-associated stem cells may be the basis of new therapeutic strategies.
